# Supplementary material for: Mechanistic insights into recruitment and regulation of the RNA helicase UPF1 in replication-dependent histone mRNA decay
Source: Nat Commun. 2026 Jan 3;17:155. doi: 10.1038/s41467-025-67991-z (PMC12775136; doi:10.1038/s41467-025-67991-z)
Supplement: Supplementary file 1 — Supplementary information [file 41467_2025_67991_MOESM1_ESM.pdf]

Supplemental information

Molecular mechanisms of recruitment, function and regulation of the RNA helicase UPF1 in replication-dependent histone mRNA decay

Alexandrina Machado de Amorim[#], Guangpu Xue[#], Wenxia He, Theresa Dittmers, Sarah Lewandowski, Cecilia Perez-Borrajero, Juliane Bethmann, Nevena Mateva, Clemens Krage, Vidhyadhar Nandana, Bernhard Loll, Tarek Hilal, Janosch Hennig, Henning Urlaub, William F. Marzluff and Sutapa Chakrabarti

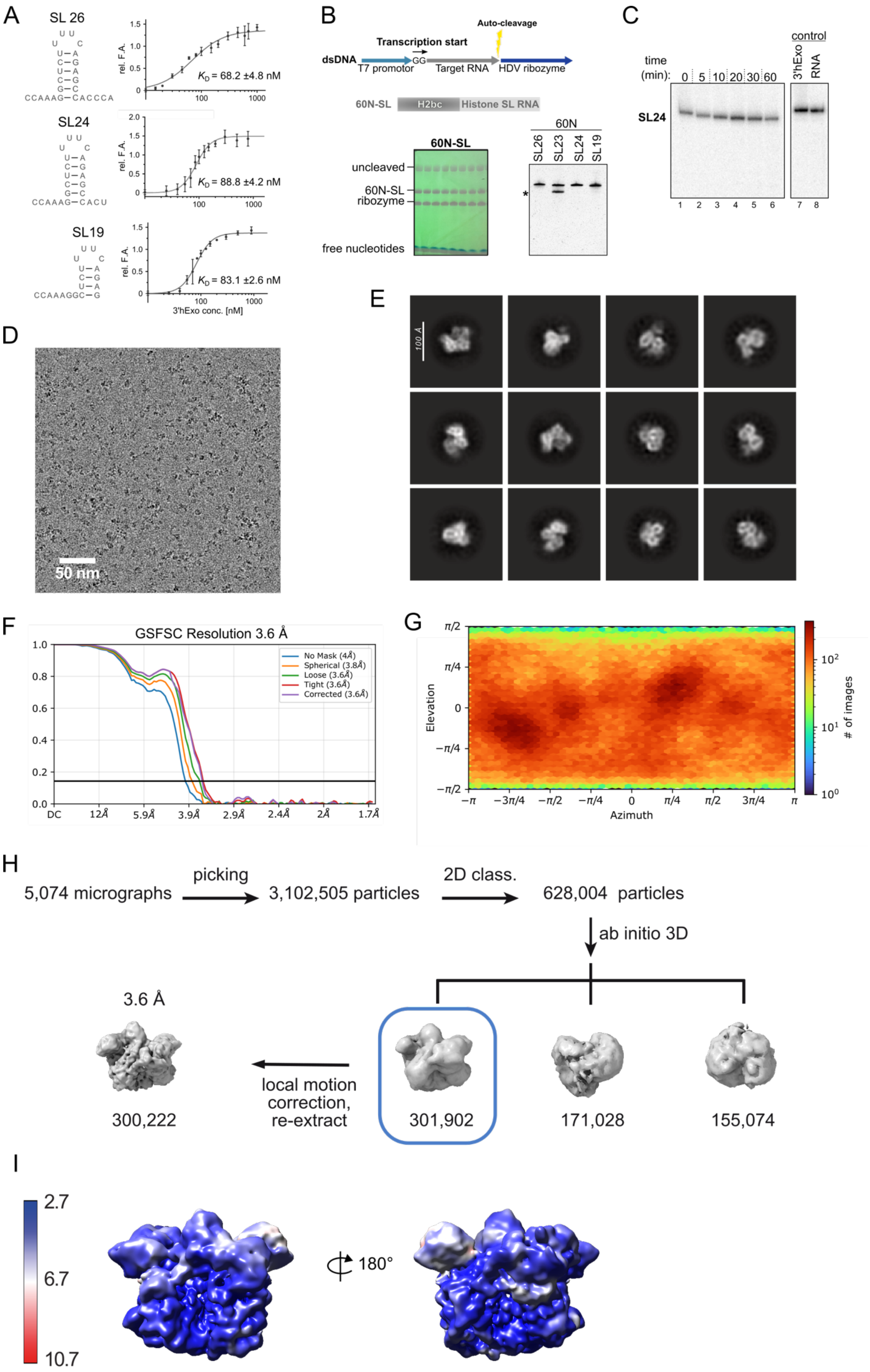

A
SL 26
K_D = 68.2 ±4.8 nM
SL24
K_D = 88.8 ±4.2 nM
SL19
K_D = 83.1 ±2.6 nM
rel. F.A.
3'hExo conc. [nM]
B
Auto-cleavage
Transcription start
dsDNA
T7 promotor
Target RNA
HDV ribozyme
60N-SL
H2bc
Histone SL RNA
60N-SL
uncleaved
60N-SL
ribozyme
free nucleotides
60N
SL26
SL23
SL24
SL19
C
control
time (min):
0
5
10
20
30
60
3'hExo
RNA
SL24
D
50 nm
E
100 Å
F
GSFSC Resolution 3.6 Å
No Mask (4Å)
Spherical (3.8Å)
Loose (3.6Å)
Tight (3.6Å)
Corrected (3.6Å)
G
Elevation
Azimuth
# of images
H
5,074 micrographs
picking
3,102,505 particles
2D class.
628,004 particles
ab initio 3D
3.6 Å
300,222
local motion correction, re-extract
301,902
171,028
155,074
I
2.7
6.7
10.7
180°

J

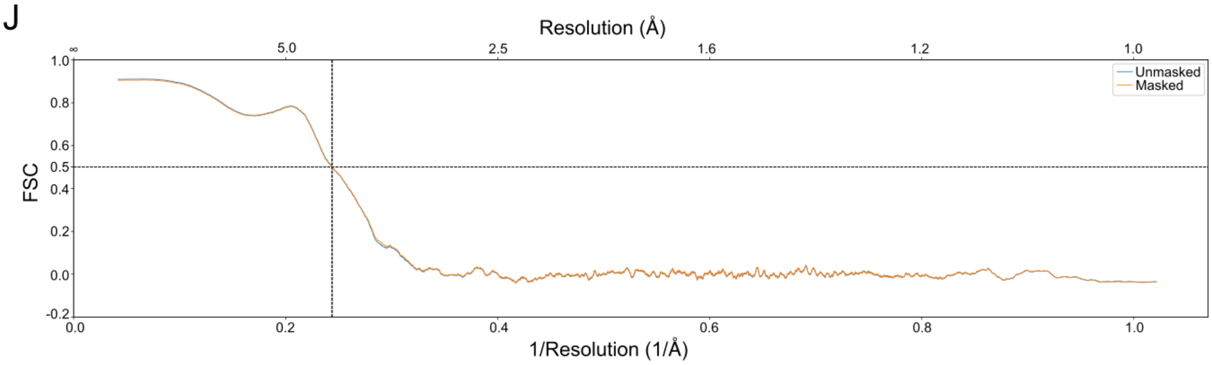

K

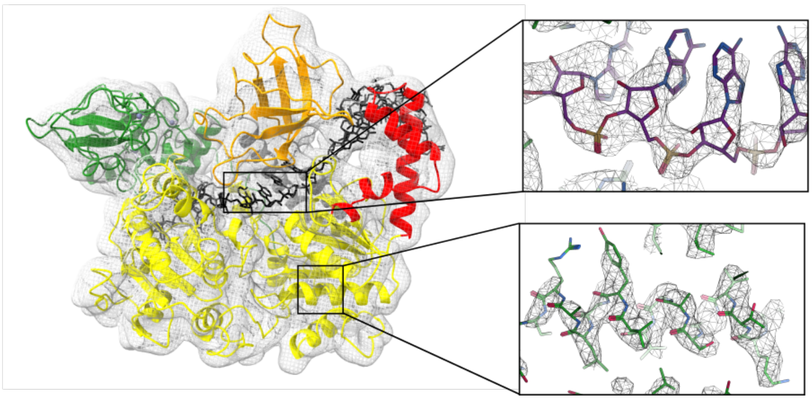

Supplementary figure 1 (related to Figure 1)

(A) Fluorescence anisotropy measurements of binding of 3’hExo to SL RNA and distinct SL degradation intermediates to derive dissociation constants (K_D). The sequence and secondary structure of each RNA is shown to the left. Data points and error bars in each plot represent the mean and standard deviation of at 3 independent experiments (n=3). The dissociation constant (K_D) reported alongside was determined by fitting the data to an equation representing one-site specific binding with Hill slope (fit denoted by a solid line). 3’hExo binds all three SL RNA substrates with comparable affinities. The binding reactions were carried out in buffer containing EDTA to inhibit exoribonuclease activity of 3’hExo and ensure that the RNA substrates are not degraded in course of the experiment.

(B) *In vitro* transcription (IVT) of RNA substrates (60N-SL) used for degradation assays.

Top: Schematic representation of the IVT template used to generate precise SL RNA species. Fusion of the HDV ribozyme to the DNA template sequence generates a precursor RNA that is spontaneously cleaved by the ribozyme to release SL RNA with precise 3’ ends. The sequence upstream of the conserved mammalian histone SL RNA is derived from the histone H2bc mRNA. Bottom: Urea-PAGE analysis of IVT reactions of the precursor 60N-SL26-HDV RNA that is autocleaved to release the mature 60N-SL26 RNA (left panel). Bands on the gel were visualized by UV-shadowing. *Cis*-cleavage by the ribozyme generates precise 3’ ends at the end of the SL for each RNA, which cannot be ensured by transcription termination of RNA

polymerase. The top, middle and lower bands correspond to the uncleaved product, the mature 60N-SL26 RNA and the ribozyme, respectively. The right panel shows a urea-PAGE analysis (visualized by ethidium bromide staining) of all RNA substrates generated using this method. SL23 shows a doublet, arising from an artefact of IVT. For biochemical studies, a single homogenous species, corresponding to the top band, is obtained by further gel purification.

(C) Time-dependent analysis of degradation of 60N-SL24 RNA by 3'hExo. The degradation reaction was initiated by addition of magnesium ions. Samples were removed from the reaction at the indicated time points and the reaction was stopped by addition of RNA loading buffer. The samples were analysed by urea-PAGE, followed by phosphorimaging. Degradation of SL24 stops at the base of the stem, after removal of 2 nucleotides from the 3' end.

(D) Representative micrograph of the UPF1-CHh:U-SL RNA complex. Particles were extracted from 5074 high-quality micrographs.

(E) Selected 2D class averages of particle images of UPF1-CHh:U-SL RNA complexes after reference-free 2D classification. A circular mask of 130 Å diameter was used in the 2D classification.

(F) Gold-standard Fourier shell correlation plot after non-uniform refinement with cryoSPARC.

(G) Viewing direction distribution plot of particles of the cryoEM reconstruction of UPF1:U-SL RNA, as obtained during refinement with cryoSPARC.

(H) Particle sorting and classification tree used for 3D reconstruction of the UPF1:U-SL RNA complex. The map selected for further high-resolution refinement is indicated by a blue box.

(I) Map of UPF1:U-SL RNA colored according to local resolution estimation, ranging from 2.7 Å to 10.7 Å.

(J) Model-to-map correlation curve, as determined during refinement using Phenix.

(K) Details of distinct regions of the UPF1:U-SL RNA model with corresponding density of the 3D reconstruction. The helicase core of UPF1 and the single-stranded RNA are well-resolved with clear features for individual sidechains and nucleotides.

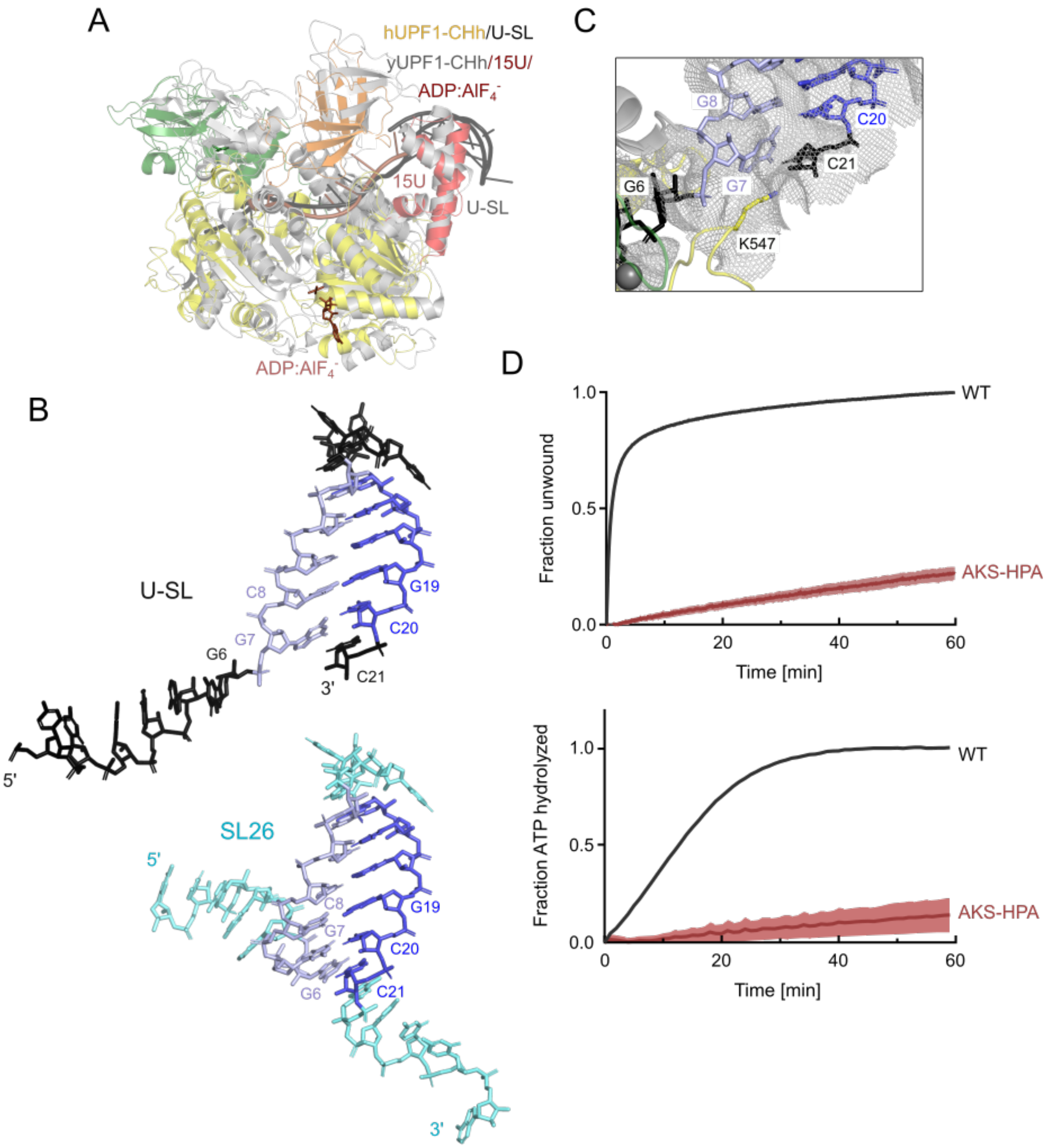

Supplementary figure 2 (related to figure 2)

(A) Superposition of the cryoEM structure of human UPF1-CHh (colored) bound to U-SL RNA (black) with the X-ray crystal structure of yeast UPF1-CHh (grey) bound to 15U RNA (dark brown) and the nucleotide ADP:AlF_4^-.

(B) Conformation of the UPF1-bound U-SL RNA derived from this study (top) and the SLBP-bound SL26 RNA, derived from the X-ray crystal structure of the SLBP:3'hExo:SL26 complex (PDB-ID 4L8R). The proteins have been omitted for clarity. Unpaired nucleotides of the RNA are shown in black for the UPF1-bound structure and in cyan for the SLBP-bound structure, while the 5' and 3' arms of the SL are shown in light and dark blue, respectively, in both structures. The nucleotides G6 and C21 and G7 and C20 are base-paired in SLBP-bound SL of the SL (bottom panel), in contrast to UPF1-bound SL (top panel).

(C) Density for nucleotides G6, G7, G8, C20 and C21 and amino acid K547 positioned at the base of the stem of SL in the UPF1:U-SL structure. Domain 1C has been removed from this view for clarity.

(D) Mutation of residues in the wedge loop (see Figure 2B) positioned at the base of the stem of U-SL RNA leads to a complete loss of UPF1 catalytic activity. Top: ATP-dependent RNA unwinding activities of wild type UPF1-Hel and UPF1 AKS-HPA on the SL RNA substrate. The individual data points, collected at 10 s intervals, represent the mean of 3 independent experiments, with technical duplicates for every experiment (n=3). Shaded areas represent the standard deviation of each measurement. Bottom: RNA-dependent ATP hydrolysis activities of UPF1-Hel variants. The relative amounts of ATP hydrolyzed by these proteins were plotted against the reaction time. Data points were collected every minute and represent the mean of 3 independent experiments, with technical duplicates for every experiment (n=3). Shaded areas represent the standard deviation of each measurement. UPF1 AKS-HPA show negligible ATPase activity, consistent with unwinding activity measurements.

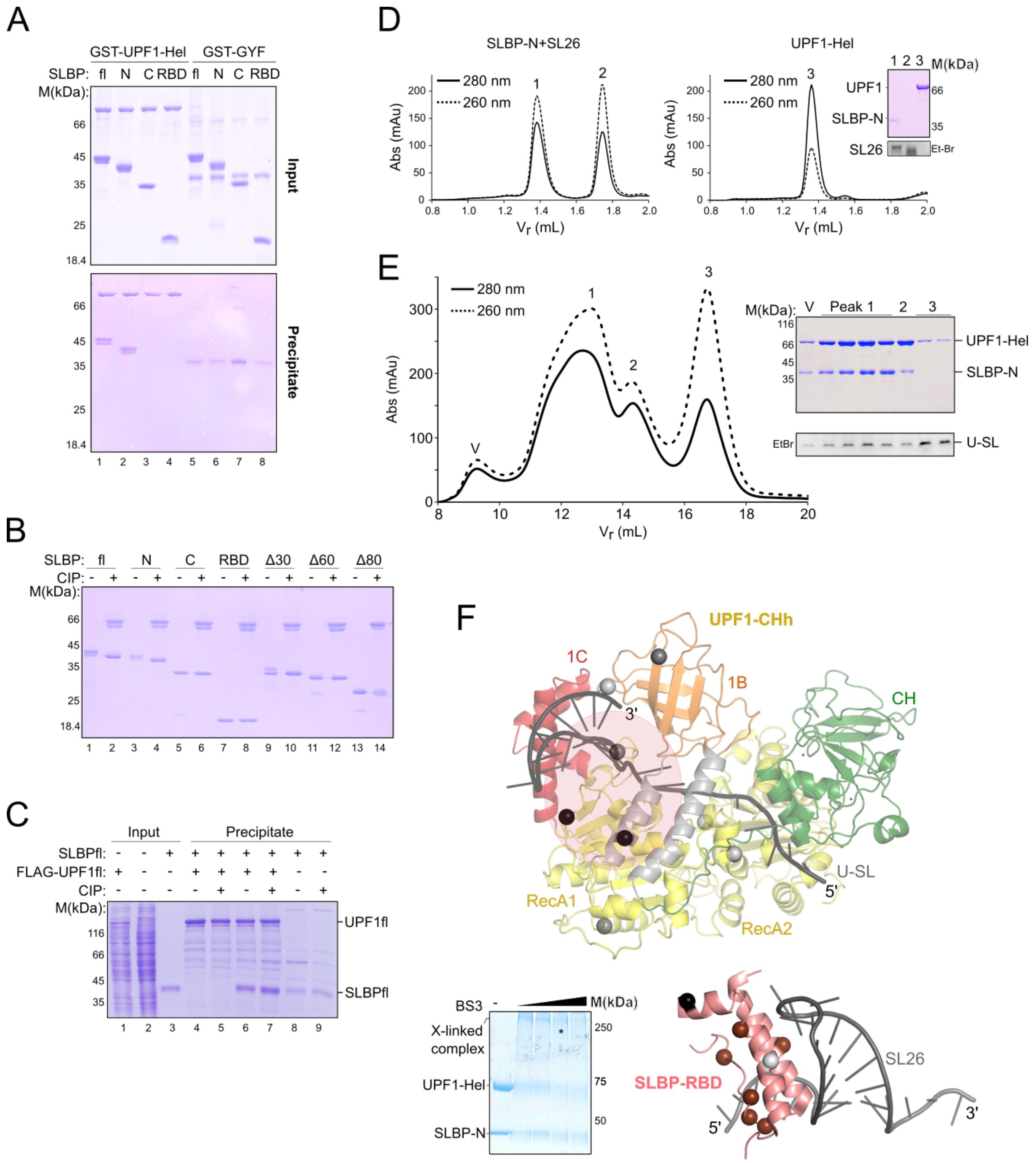

Supplementary figure 3 (related to Figure 3)

(A) The complete SDS-PAGE gel corresponding to Figure 3B, including negative controls using GST-GYF as a bait. SLBP variants used in this experiment are shown in Figure 3B. This and all other GST-pulldowns were independently performed three times with similar results.

(B) SDS-PAGE analysis of phosphatase-treatment of SLBP proteins. Compaction of the bands corresponding to SLBPfl, SLBP-N, SLBPΔ30 and SLBPΔ60 upon CIP-treatment and the concomitant increase in mobility on SDS-PAGE suggests that these proteins were phosphorylated upon expression in insect cells. SLBP-C and RBD did not show any change in

migration pattern on SDS-PAGE upon CIP treatment. This experiment was independently performed twice with similar results.

(C) Co-precipitation of SLBPfl with Flag-UPF1fl expressed in HEK293 cells. Extracts were treated with calf intestinal phosphatase (CIP) to generate unphosphorylated UPF1fl protein. SLBPfl efficiently co-precipitates with CIP-treated and mock-treated UPF1, indicating that phosphorylation of UPF1 does not influence its binding to SLBP. This experiment was independently performed three times with similar results.

(D) Analytical SEC of an SLBP-N:SL26 complex (left panel) and UPF1-Hel (middle panel). The corresponding SDS- and urea-PAGE analyses of the peak fractions are shown on the right. Appearance of a lower retention volume peak (1.25 mL) upon addition of SL26 RNA to the UPF1:SLBP protein mixture (Figure 3C) indicates formation of a stable UPF1:SLBP complex only when SLBP is bound to the histone SL RNA. The SEC analysis was performed twice.

(E) Preparative SEC for the reconstitution of a ternary complex of UPF1-Hel, SLBP-N and U-SL RNA for crosslinking mass-spectrometry (CLMS). The chromatogram of the SEC run and the corresponding SDS- and urea-PAGE analyses are shown on the left and right, respectively. Peak fractions of peak 1 were pooled for CLMS analysis.

(F) Top panel: Structure of the UPF1-CHh:U-SL RNA complex with the position of residues crosslinked to SLBP highlighted as spheres. The color scheme of the spheres (black, dark grey and light grey) corresponds to the color of the lines denoting interlinks in Figure 3D. The pink shaded region denotes the site where SLBP would bind the SL RNA, prior to its distortion by UPF1.

Bottom left panel: SDS-PAGE analysis of the UPF1-Hel:SLBP-N:U-SL complex crosslinked using increasing concentrations of BS3. The uncrosslinked complex is shown for comparison. The band denoted by * was excised from the gel for mass spectrometric analysis. The cross-linking reaction was performed three times and measurements were conducted in triplicate each time.

Bottom right panel: structure of SLBP:SL26-RNA (derived from the X-ray crystal structure of the SLBP/3’hExo/SL26 complex, PDB-ID 4L8R), with the position of the residues within the SLBP-RBD that crosslink to UPF1 highlighted as spheres (black, dark grey and light grey, as above). Lysine residues within the SLBP-RBD that were not crosslinked to UPF1 are shown as dark brown spheres.

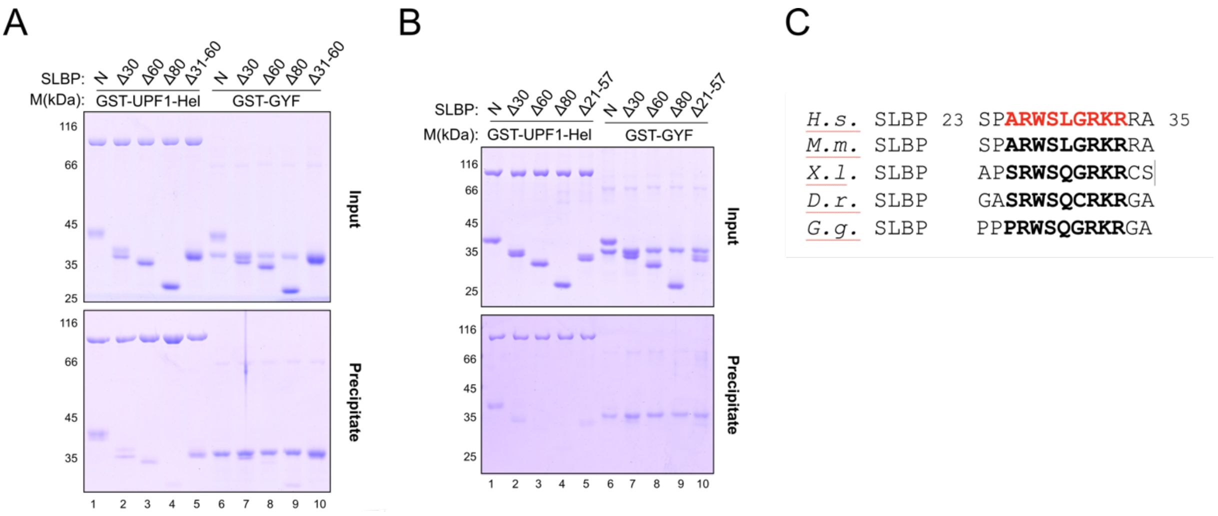

Supplementary figure 4 (related to Figure 4)

(A) GST-pulldown assay of GST-UPF1-Hel (and GST-GYF) with SLBP N-terminal truncations including SLBPΔ31-60, as in Figure 4A of main text.

(B) The complete SDS-PAGE gel corresponding to Figure 4A, including negative controls using GST-GYF as a bait. SLBP variants used in this experiment are shown in Figure 4A.

(C) Sequence alignment of the putative UPF1-binding motif located within the N-terminal IDRs of human (*H.s.*), mouse (*M.m.*), aquatic frog (*X.l.*), zebrafish (*D.r.*) and chicken (*G.g*) SLBP proteins. Residue numbers for human SLBP are indicated.

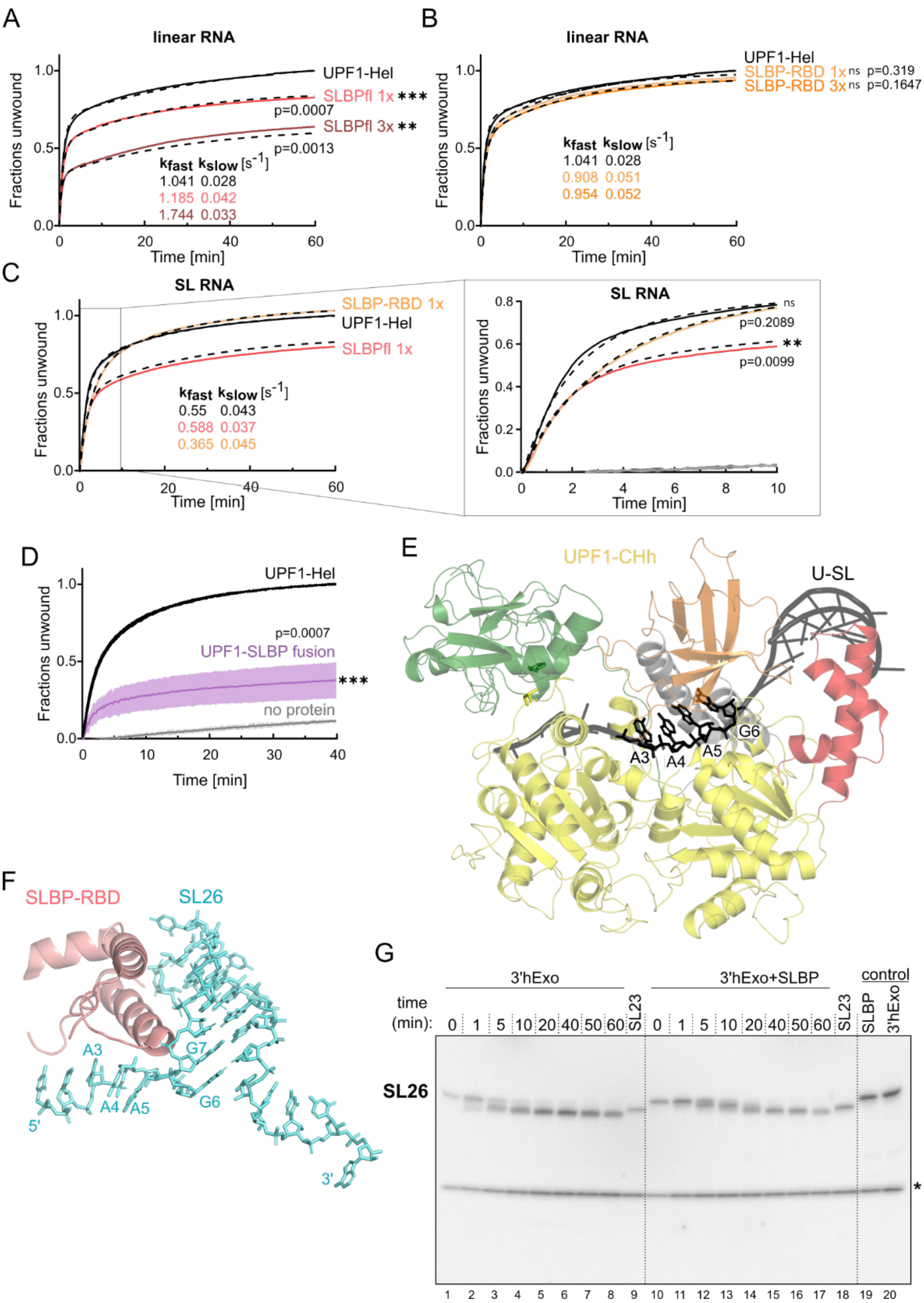

Supplementary figure 5 (related to Figure 5)

(A-C) Unwinding assays of Figures 5(A-C) fitted to a two-state decay model. The experimental data are shown as colored lines while model fits are denoted as dashed lines. First-order rate constants, k_{fast} and k_{slow}, derived from each fit are indicated in the plots. Statistical significance of the differences among unwinding reactions (denoted by asterisks) were determined by unpaired two-tailed t-tests. Individual p-values are shown in every case. ** = very significant,

$p \leq 0.01$; *** = extremely significant, $p \leq 0.001$; ns= not significant, $p > 0.05$. n=3 independent experiments.

(D) Unwinding activity of UPF1-SLBP fusion on the SL-RNA substrate. Statistical analysis was carried out as described above. Strong inhibition was observed upon linking the SLBP N-IDR to the UPF1 helicase core. n= 3 independent experiments.

(E) Structure of the UPF1-CHh:U-SL RNA complex, with the positions of nucleotides A3-G6 within the RNA-binding channel of UPF1 highlighted as sticks. The rest of the U-SL RNA is shown in cartoon form.

(F) X-ray crystal structure of the SLBP:3'hExo:SL26 RNA (PDB 4L8R), with the SLBP-binding nucleotides A3-G7 highlighted. 3'hExo is omitted from this figure. Comparison of this structure with (E) above shows that the nucleotides of SL26 recognized by SLBP are no longer available for binding this protein when in complex with UPF1.

(G) Time-dependent analysis of degradation of 60N-SL26 RNA by 3'hExo in the absence (lanes 1-8) and presence (lanes 10-17) of SLBP. The experiment was carried out as described in Supplementary figure 1C above. Degradation of SL26 stops at the base of the stem, after removal of 5 nucleotides from the 3' end. Addition of SLBP slows down degradation of SL26, acting as a roadblock for 3'hExo (compare lanes 3 and 12). * denotes a labelled DNA loading control used in this experiment. The degradation analysis was independently performed 6 times with identical results.

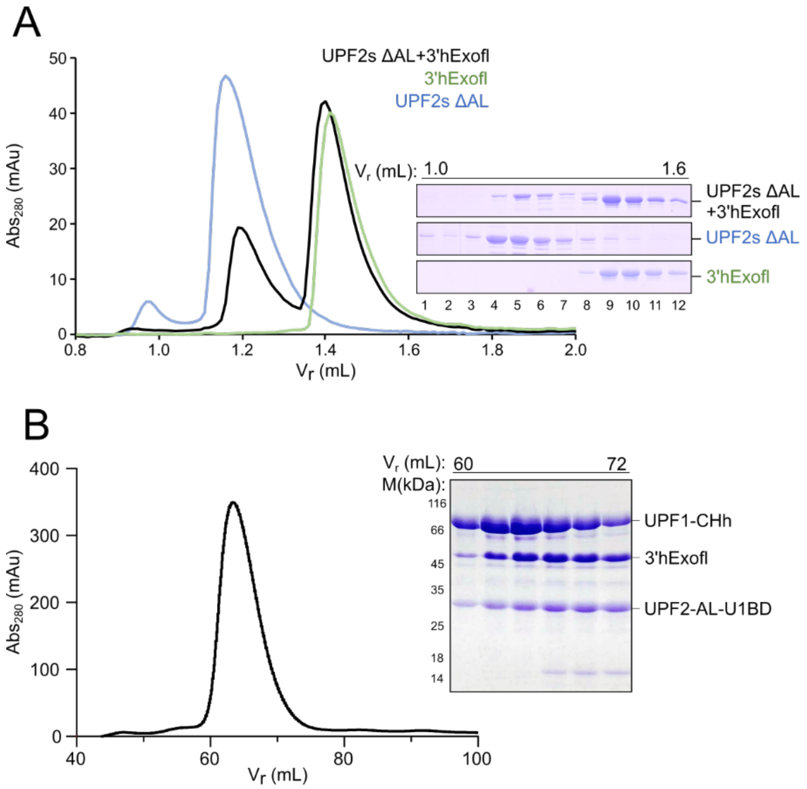

Supplementary figure 6 (related to Figure 6)

(A) Analytical SEC of 3'hExofI and a UPF2 protein lacking the acidic linker (UPF2ΔAL). No complex formation is observed, confirming that the acidic linker of UPF2 is necessary for binding to 3'hExo. This analysis was performed twice with similar results.

(B) SEC and corresponding SDS-PAGE analysis depicting formation of a stable ternary complex of UPF1-CHh, UPF2s and 3'hExo. The exclusion volume of the column is 40 mL. The complex reconstitution was performed 4 times with similar results.

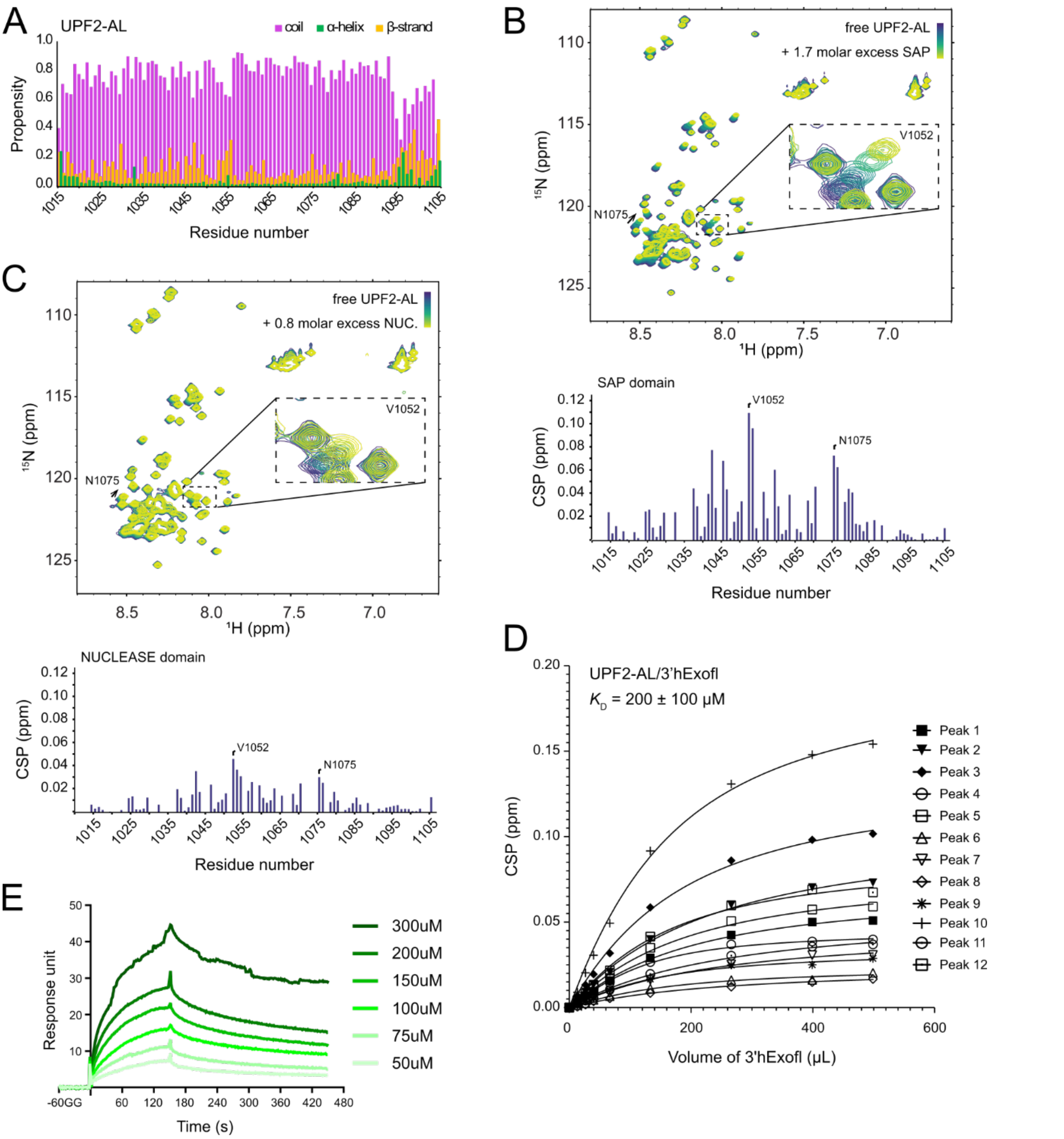

Supplementary figure 7 (related to Figure 7)

(A) Secondary structure prediction of UPF2-AL based on NMR chemical shifts. The acidic linker appears to be intrinsically disordered, as expected for a low-complexity sequence.

(B-C) ^{1}H-^{15}N-HSQC NMR titration experiments of ^{15}N-labeled UPF2s with increasing concentrations of 3'hExo-SAP (B) and Nuclease (C) domains. The spectrum of free UPF2-AL is in blue while those recorded in presence of increasing concentrations of 3'hExo proteins are in progressively lighter shades of green. The insets show a zoomed-in view of residue V1052 of UPF2-AL that shows the largest chemical shift perturbations (CSP) upon addition of 3'hExo-SAP and Nuclease proteins, as observed with 3'hExofl (Figure 7B). The histograms of the CSPs of UPF2-AL upon titration of 3'hExo-SAP and Nuclease are plotted against the UPF2-

AL protein sequence and shown below each spectrum. The patterns of CSPs obtained upon titration of the SAP and Nuclease subdomains are very similar, suggesting that they bind the same stretch of residues of UPF2.

(D) Plot of CSP vs. volume of 3'hExofl added to derive the dissociation constant (K_D) of the UPF2-AL:3'hExo interaction. To ensure that the concentration of UPF2-AL protein could be accurately determined and its purity assessed by SDS-PAGE analysis, a modified version of the UPF2 acidic linker was designed, spanning residues 1024-1085 and containing three lysine residues and one tryptophan at the N- and C-termini, respectively.

(E) Binding affinity of 3'hExofl for UPF2-AL1 as measured by surface plasmon resonance. 3'hExofl was immobilized on a CM5 chip and UPF2-AL1 was injected at concentrations ranging from 50-300 μM. Binding constants were derived from 3 independent experiments by fitting the data to a 1:1 binding model.

Supplementary Table 1

DNA oligonucleotide templates for cloning		
**Oligo Name**	**Description**	**Sequence (5'→3')**
oAA100	Mutation A546H in UPF1 fwd	GTGCGCCTCTGCCACAAGAGCCGTGAG
oAA101	Mutation A546H in UPF1 rev	CTCACGGCTCTTGTGGCAGAGGCGCAC
oAA102	Mutation K547P and S548A in UPF1 fwd	GTGCGCCTCTGCCACCCGGCCCGTGAGGCCATCG
oAA103	Mutation K547P and S548A in UPF1 rev	CGATGGCCTCACGGGCCGGGTGGCAGAGGCGCAC
oAA130	SLBP part of UPF1-SLBP	CCGTCCCCCGCGCGATGGA
oAA131	UPF1 part of UPF1-SLBP	GCTGAACTGCATGAGGCTCtcacgcag
oES144	UPF1(295) LIC fwd	CCAGGGAGCAGCCTCGATGCGGTACGAGGACG
oES145	UPF1(914) LIC rev	gcaaagcaccggcctcgttagctgaactgcatgag
oSC45	3'hExo (1) 3C_LIC fwd	CCAGGGGCCCGACTCGATGgaggatccacagagtaaag
oSC46	3'hExo (55) 3C_LIC fwd	CCAGGGGCCCGACTCGATGacctccagtgcgagtgac
oSC47	3'hExo (349) 3C_LIC rev	CAGACCGCCACCGACTGCTTActttctaaaatgtggc
oSC177	3'hExo (124) 3C_LIC fwd	CCAGGGGCCCGACTCGATGgacagttattatgactac
oSC179	3'hExo (124) 3C_LIC rev	CAGACCGCCACCGACTGCTTAgtcagcaaaattgctctc
oSC239	UPF2(∆1055-1104) fwd	gaagtaaatgagGGTAGTGCTGGATCTGCAgtaccttgtgtag aag
oSC240	UPF2(∆1055-1104) rev	cacaaggtacTGCAGATCCAGCACTACCctcatttacttcact ttc
oSC276	UPF2(1015) 3C_LIC fwd	CCAGGGGCCCGACTCGATGaatgacaaagactc
oSC278	UPF2(1106) 3C_LIC rev	CAGACCGCCACCGACTGCTTAaggtacatgcttaagtcc

DNA oligonucleotide templates for IVT		
**Oligo Name**	**Description**	**Sequence (5'→3')**
oAA28	60N-SL+HDV oligo fwd	CTAATACGACTCACTATAGGCCGTCACCAAGTAC
oAA54	60N-SL26+HDV oligo rev	GTCCCATTCGCCATCGCGAACGATGTTGCCCACCGGCCGCCAG CGAGGAGGCTGGGACCATGGCCGGCTGGGTGGCTCTGAAAAGA GCCTTTGGGGTTAG
oAA55	60N-SL19+HDV oligo rev	GTCCCATTCGCCATCGCGAACGATGTTGCCCACCGGCCGCCAG CGAGGAGGCTGGGACCATGGCCGGCCTCTGAAAAGAGCCTTTG GGGTTAG
oAA26	60N-SL23+HDV oligo fwd	CTAATACGACTCACTATAGG CCGTCACCAAGTACACCAGCTCCAAGTAAACATTCCAAGTAAG CGTCTTAACACCTAACCCCAAAGGCTCTTTT
oAA27	60N-SL23+HDV oligo rev	CTAACCCCAAAGGCTCTTTTCAGAG CCAC GCCGGCCATGGTCCCAGCCTCCTCGCTGGCGGCCGGTGGGCAA CATCGTTCGCGATGGCGAATGGGAC
oAA118	60N-SL24+HDV oligo rev	GTCCCATTCGCCATCGCGAACGATGTTGCCCACCGG CCGCCAGCGAGGAGGCTGGGACCATGGCCGGCAGTG GCTCTGAAAAGAGCCTTTGGGGTTAGGT
oAA79	SL RNA unwinding assay oligo fwd	CTAATACGACTCACTATAGGGACACAAAACAAAAGACAAAAAC ACAAAACAAAAGACAAAAACACAAAACAAAAG

oAA80	SL RNA unwinding assay oligo rev	AGCTAGTTGTACGCACACGGTGGCTCTGAAAAGAGCCTTTGGCTTTTTGTCTTTTGTTTTGTGTTTTTGTCTTTT
oAA81	Linear RNA unwinding assay oligo rev	AGCTAGTTGTACGCACACGGTAATTTGGCTTTTTGTCTTTTGTTTTGTGTTTTTGT

RNA substrates generated by IVT

Name	Description	Sequence (5'→3')
60N-SL26	upstream H2bc sequence(60 nt) + histone SL 26mer (full-length)	GGCCGUCACCAAGUACACCAGCUCCAAGUAAACAUUCCAAGUAAGCGUCUUAACACCUAACCCCAAAGGCUCUUUUCAGAGCCACCCA
60N-SL23	upstream H2bc sequence(60 nt) + histone SL 23mer (FL-3 nt)	GGCCGUCACCAAGUACACCAGCUCCAAGUAAACAUUCCAAGUAAGCGUCUUAACACCUAACCCCAAAGGCUCUUUUCAGAGCCACU
60N-SL24	upstream H2bc sequence(60 nt) + histone SL 24mer (FL-3 nt+1 U)	GGCCGUCACCAAGUACACCAGCUCCAAGUAAACAUUCCAAGUAAGCGUCUUAACACCUAACCCCAAAGGCUCUUUUCAGAGCCAC
60N-SL19	upstream H2bc sequence(60 nt) + SL 19mer (FL-7nt)	GGCCGUCACCAAGUACACCAGCUCCAAGUAAACAUUCCAAGUAAGCGUCUUAACACCUAACCCCAAAGGCUCUUUUCAGAG
SL UWA	for unwinding assay, SL 24mer (FL-2 nt), downstream hybridization site (18 nt), upstream UPF1 translocation site (63 nt)	GGGACACAAAACAAAAGACAAAAACACAAAACAAAAGACAAAAACACAAAACAAAAGACAAAAAGCCAAAGGCUCUUUUCAGAGCCACCGUGUGCGUACAACUAGCU
Linear UWA	for unwinding assay, ss ctrl substrate, downstream hybridization site (18 nt), upstream UPF1 translocation site (63 nt)	GGGACACAAAACAAAAGACAAAAACACAAAACAAAAGACAAAAACACAAAACAAAAGACAAAAAGCCAAAUUACCGUGUGCGUACAACUAGCU

Synthetic RNAs

Name	Sequence (5'→3')
12U-26SL	UUUUUUUUUUUUCCAAAGGCUCUUUUCAGAGCCACCCA
5‘ 6-FAM-SL26	6-FAM/CCAAAGGCUCUUUUCAGAGCCACCCA
5‘ 6-FAM-SL24	6-FAM/CCAAAGGCUCUUUUCAGAGCCACU
5‘ 6-FAM-SL19	6-FAM/CCAAAGGCUCUUUUCAGAG
5‘ Alexa488-labeled DNA probe	Alexa488/AGCTAGTTGTACGCACAC
3‘ Black Hole Quencher 1- conjugated DNA trap	GTGTGCGTACAACTAGCT/3BHQ_1- 3’

Primer and sgRNA Sequences

Name	Sequence (5'→3')
SLBP-gF1	CCG GGG ACG CGG TCG GCT GGG CAC
SLBP-gR1	AAA CGT GCC CAG CCG ACC GCG TCC
SLBP-gF2	CCG GCG AGG GAG CGC GTG CCC CGT
SLBP-gR2	AAA CAC GGG GCA CGC GCT CCC TCG
SLBP-gF3	CCG GTC GCG GTG CCG GGA TCG GTC

SLBP-gR3	AAA CGA CCG ATC CCG GCA CCG CGA
SLBP-gF4	CCG GGG AGC CCG CGG CCT CGT CAA
SLBP-gR4	AAA CTT GAC GAG GCC GCG GGC TCC
SLBP-tF	GTTGTAAGGCGGTCCCGAA
SLBP-tR	GTTCAGCTGACTCCCATCCT
SLBP-RT-F	ATCAGAGCCGCTGCGACGGTGACGCCA
SLBP-RT-R	TCAGAACTTCCTGATGATGA

Supplmentary table 2: Filtered Pivot Table

Row Labels	Protein1	Protein2	Pos1	Pos2	Count of Peptide	Max of -logscore
**Inter-Protein**	**hSLBP_NT**	hSLBP_NT	**97**	97	6	2.550853272
			**128**	128	11	8.667823003
			**186**	186	11	4.944490941
		hUPF1_helicasecore	**97**	271	4	7.693135967
				351	7	10.75669252
				601	3	8.979255564
			**128**	271	3	8.971394997
				351	18	20.05567573
			**170**	351	3	8.930571312
			**186**	271	6	5.846776789
				351	6	7.485890615
	**hUPF1_helicasecore**	hSLBP_NT	**63**	186	7	7.843314888
			**73**	97	3	8.952763695
				186	6	11.506198
			**99**	128	4	7.01637484
			**152**	97	7	16.68064302
				120	9	13.10739835
				128	7	22.69822734
				186	11	12.28771093
			**204**	97	3	4.438418533
				128	13	13.38673657
			**351**	120	9	6.344130606
				186	8	4.32720469
			**401**	128	7	19.45144181
			**601**	128	3	7.983114513
				186	8	11.02552087
			**620**	186	3	9.111367963
**Intra-Protein**	**hSLBP_NT**	hSLBP_NT	**97**	120	19	7.474208072
				128	14	9.593347939
				170	7	8.391611657
				186	14	5.057580183
			**128**	120	27	7.483458545
				170	9	9.071499684
				186	4	8.662481239
			**139**	128	11	15.27583067
			**170**	128	3	4.5615806
				186	10	3.491233739
			**176**	186	11	7.945721412
			**186**	120	9	4.377489431
	**hUPF1_helicasecore**	hUPF1_helicasecore	**35**	152	12	14.39486429
			**45**	271	6	2.691477844
			**73**	45	17	12.63488601
			**140**	99	15	10.81427997
				271	7	2.691980557
			**152**	351	46	13.03678204
			**351**	271	12	6.459305471
			**499**	506	11	4.202590696
			**506**	499	10	9.284390874
			**601**	351	3	2.488910988
			**617**	601	8	2.010320147
**(blank)**	**(blank)**	(blank)	**(blank)**	(blank)		
**Grand Total**					**471**	**22.69822734**

Supplementary Methods

CIP treatment of SLBP proteins

Equal amounts (2 μg) of each SLBP variant were treated with 5 U of calf intestinal phosphatase (CIP) for 30 minutes at 30 °C. The reactions were quenched by addition of 4X SDS-PAGE buffer. Mock-treated (-) and CIP-treated (+) samples were analysed by SDS-PAGE and visualized by staining with Coomassie Brilliant Blue.

Expression and immunoprecipitation of UPF1fl from HEK293 cells

To determine the impact of UPF1 phosphorylation on the the UPF1-SLBP interaction, a coimmunoprecipitation (co-IP) was performed with Flag-tagged full-length (fl) UPF1 expressed in HEK293 cells and purified SLBPfl protein. For expression of Flag-UPF1fl, HEK293 cells were seeded in DMEM high glucose medium (Biowest) supplemented with 10% fetal bovine serum (FBS, Bio&Sell) at a density of 0.4 x10^6/mL, 24h prior to transfection. Each 10 cm plate of HEK293 cells were transfected with 10 µg plasmid encoding Flag-UPF1fl using polyethyleneimine (Polysciences Inc). Cells were harvested 48h post-transfection; untransfected cells were used as a control for IP. The harvested cells were lysed in NET-G buffer (50 mM Tris–HCl pH 7.5, 150 mM NaCl, 10% Glycerol, and 0.1% NP40). Half of the cell lysate was treated with Quick CIP (NEB) and the other half was subjected to mock treatment (the same volume of buffer was added). The CIP-treated and mock-treated samples were incubated at 30 °C for 30 min. Following the treatment, Flag-UPF1fl was immunoprecipitated from the lysate using the anti-Flag M2 affinity agarose (Merck). The resin was extensively washed with co-IP buffer (20 mM Hepes pH 7.5, 70 mM NaCl, 10% Glycerol, 1 mM $MgCl_2$, and 1 µM $ZnCl_2$), followed by addition of 10 µg of purified SLBPfl, where indicated. The binding reactions were further incubated for 2 h at 4 °C. The beads were washed again with co-IP buffer and the captured proteins were eluted in 25 µl of 2x SDS-PAGE sample loading buffer lacking DTT. Inputs and eluates were analyzed by SDS-PAGE and Coomassie staining.

RNA-dependent ATPase activity assay

RNA-stimulated ATPase assays were performed using the EnzCheck^{TM} Phosphate Assay Kit (ThermoFisher). For each replicate, 40 µl 2-amino-6-mercapto-7-methylpurine ribonucleoside, 4 µl purine nucleoside phosphorylase (PNP), 20 pmol of the respective UPF1 protein and 2 µg of poly(U) RNA (Sigma) were mixed in 1x ATPase reaction buffer (50 mM MES buffer pH 6.5, 50 mM potassium acetate, 5 mM magnesium acetate) to a final volume of 150 µl, incubated for 30 min at 25 °C and added to a flat-bottomed, 96-well plate (Greiner). Negative controls contained 1x ATPase buffer instead of protein or RNA. 50 µl of 4 mM ATP in 1x ATPase reaction buffer was added to each sample to start the reaction using the injector module. Absorbance was measured at 360 nm using a Spark multimode microplate reader

(Tecan). Absorbance was monitored over a period of 30 minutes at 60 s intervals. The plate was shaken every minute in an orbital shaker to ensure proper mixing. Technical duplicates were run for each condition. Values were baseline-corrected by subtracting the initial absorbance reading immediately following the addition of ATP. This was then normalized to the baseline-corrected maximum absorbance value for each data set. The data shown are a mean of 3 independent experiments. The shaded regions represent standard deviation across the 3 independent experiments. Values were averaged and plotted using the Prism software (GraphPad).

Surface Plasmon Resonance

Surface plasmon resonance experiments were performed on a Biacore Upgrade instrument (Biacore^{TM} X100) at 25 °C. Research grade carboxymethyldextran (CM5) chips were purchased from Cytiva. *N*-hydroxysuccinimide (NHS), *N*-ethyl-*N′*-(3-dimethylaminopropyl) carbodiimide hydrochloride (EDC) and ethanolamine were also procured from Cytiva. HBS-EP (10 mM HEPES pH 7.5, 125 mM NaCl, 3 mM EDTA, 0.005% Biacore Surfactant P20) and 10 mM sodium acetate buffer were prepared in lab. The CM5 sensor Chip was soaked in HBS-EP buffer overnight at room temperature before use. Then the chip was dried and loaded to the Biacore chamber. 3'hExofl was diluted to 1.2 µg/mL with 10 mM sodium acetate pH 5.0 buffer and injected in pulses on an EDC/NHS activated CM5 Biacore chip until 1900 response units (RU) were immobilized on the CM5 Biacore chip. Remaining activated carboxyl groups were quenched with Ethanolamine. The UPF2-AL protein, ranging from 300 µM to 50 µM, were then applied onto the chip and injected for a contact time of 150 s. 5 M NaCl was used as a regeneration solution. All experiments were repeated three times. The k_{on}, k_{off} and K_D, were determined by analyzing the kinetic data by a global fit to a 1:1 binding model with the BIA evaluation software (Biacore, Cytiva).
