# Supplementary material for: Mechanistic insights into recruitment and regulation of the RNA helicase UPF1 in replication-dependent histone mRNA decay
Source: Nat Commun. 2026 Jan 3;17:155. doi: 10.1038/s41467-025-67991-z (PMC12775136; doi:10.1038/s41467-025-67991-z)

The regions of the gels/blots highlighted by black boxes in this and all other figures indicate the part of the gels used to generate the final figures. Figure numbers in the source data file refer to the corresponding figures in the main text or supplementary information.

Figure 1C

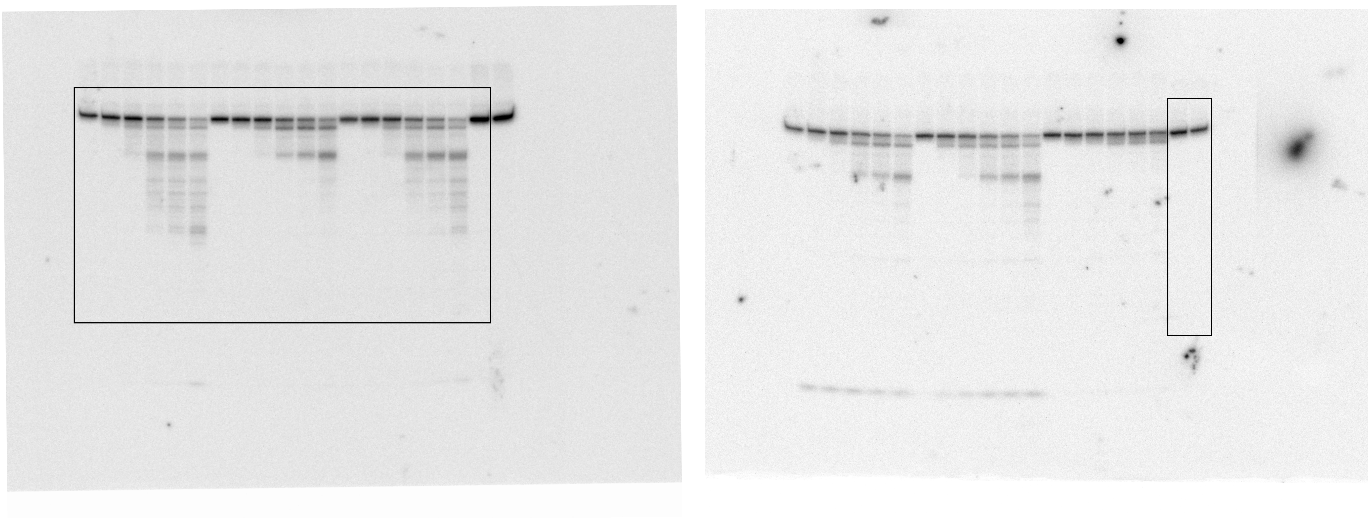

Figure 2C

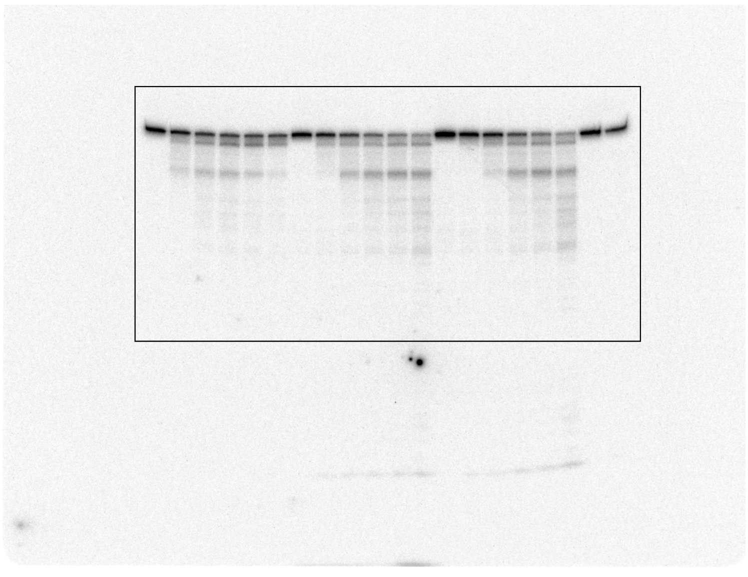

Figure 3A

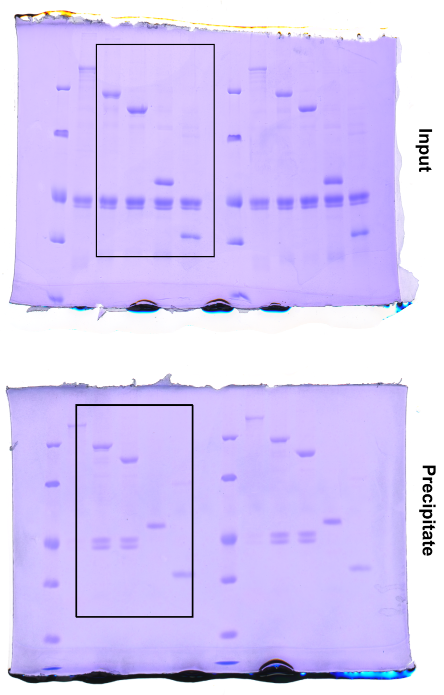

Figure 3B

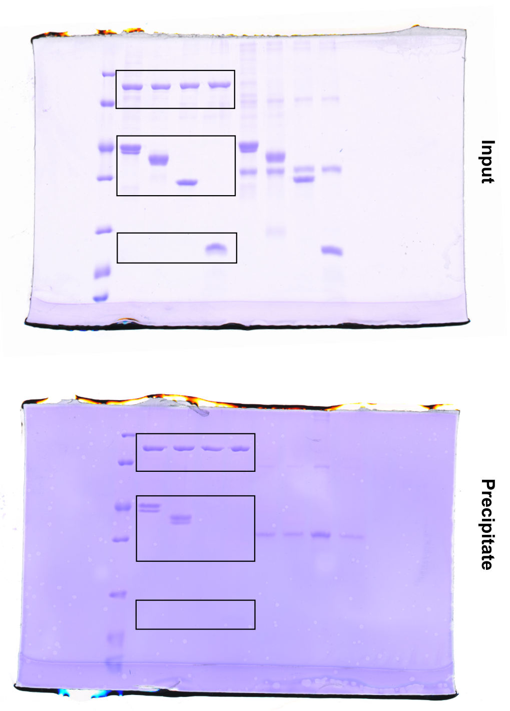

Figure 3C

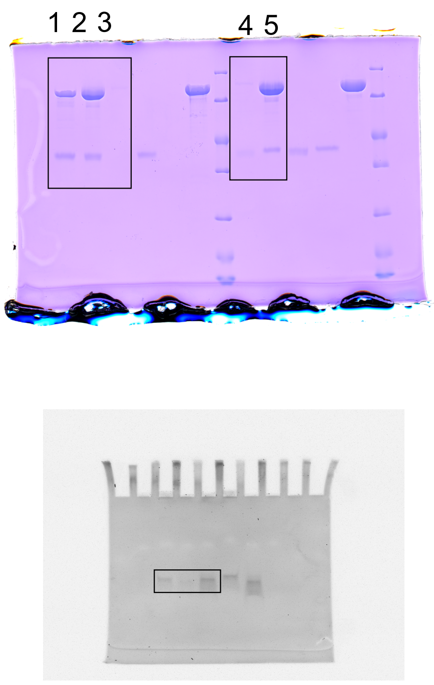

Figure 4A

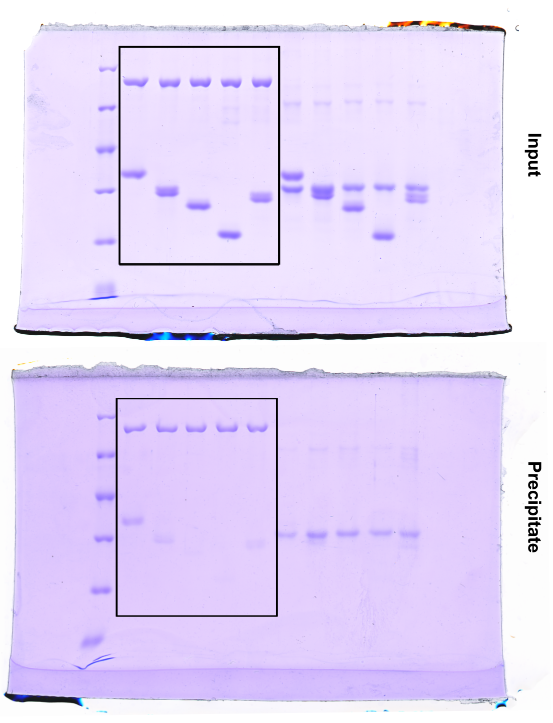

Figure 4B

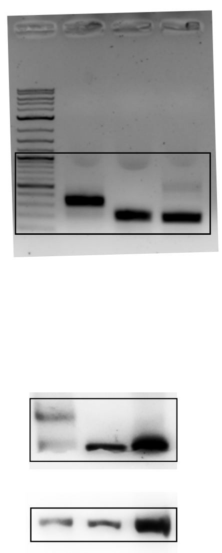

Figure 4C

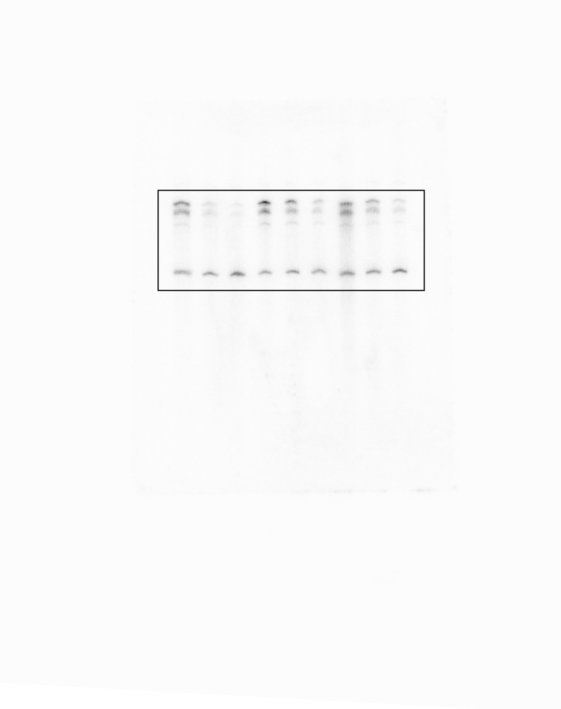

Figure 6D

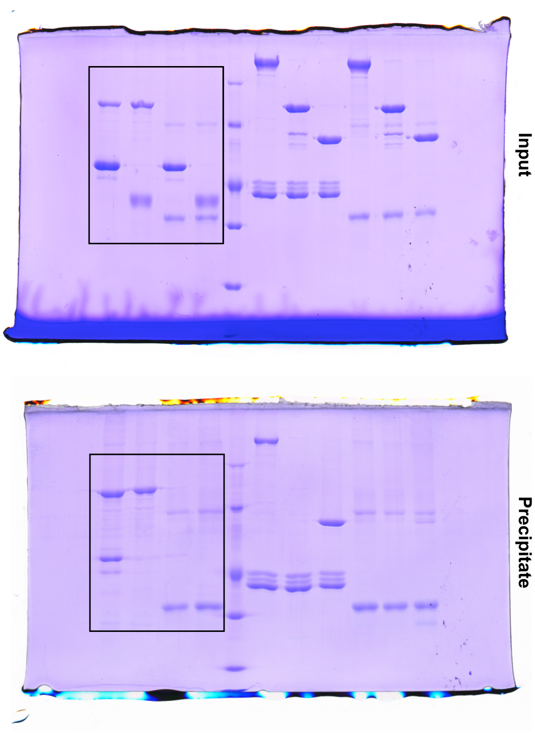

Figure 6E

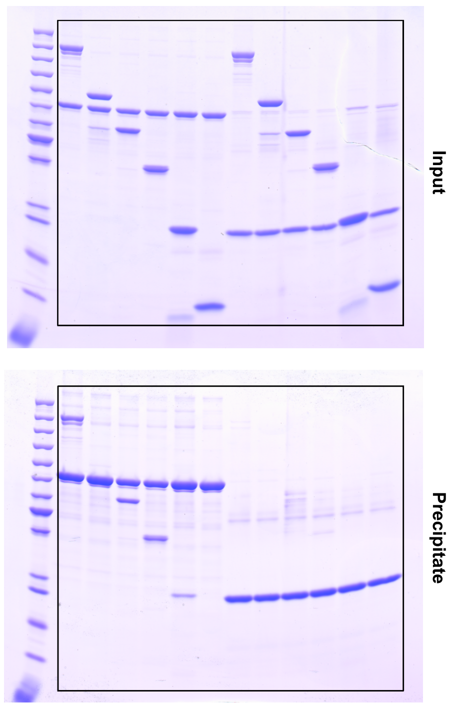

Figure 6F

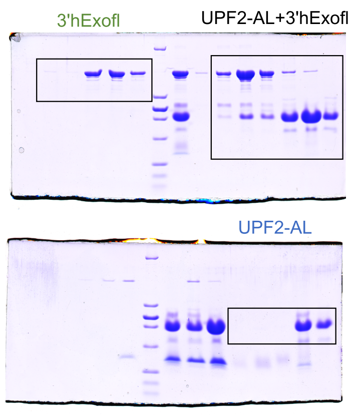

Figure 7A

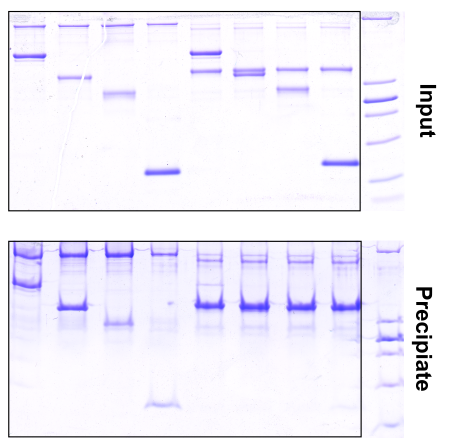

Supplement: Supplementary file 4 — Source Data [file 41467_2025_67991_MOESM4_ESM.zip › Sourcedata.pdf]
