# Supplementary material for: Mechanistic insights into recruitment and regulation of the RNA helicase UPF1 in replication-dependent histone mRNA decay
Source: Nat Commun. 2026 Jan 3;17:155. doi: 10.1038/s41467-025-67991-z (PMC12775136; doi:10.1038/s41467-025-67991-z)

The regions of the gels/blots highlighted by black boxes in this and all other figures indicate the part of the gels used to generate the final figures. Figure numbers in the source data file refer to the corresponding figures in the main text or supplementary information.

Sup. Figure 1B

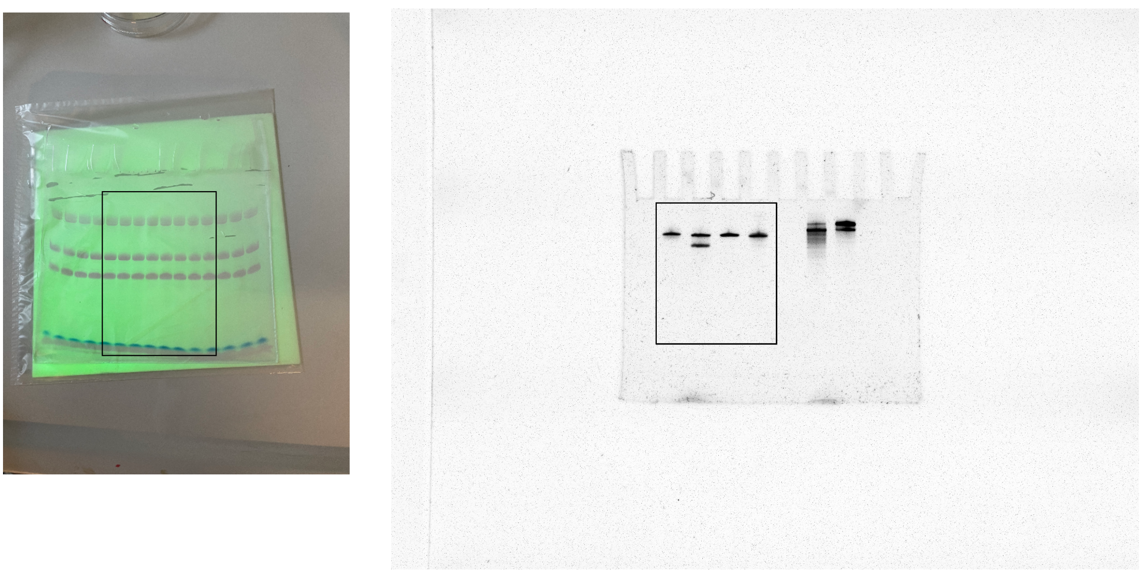

Sup. Figure 1C

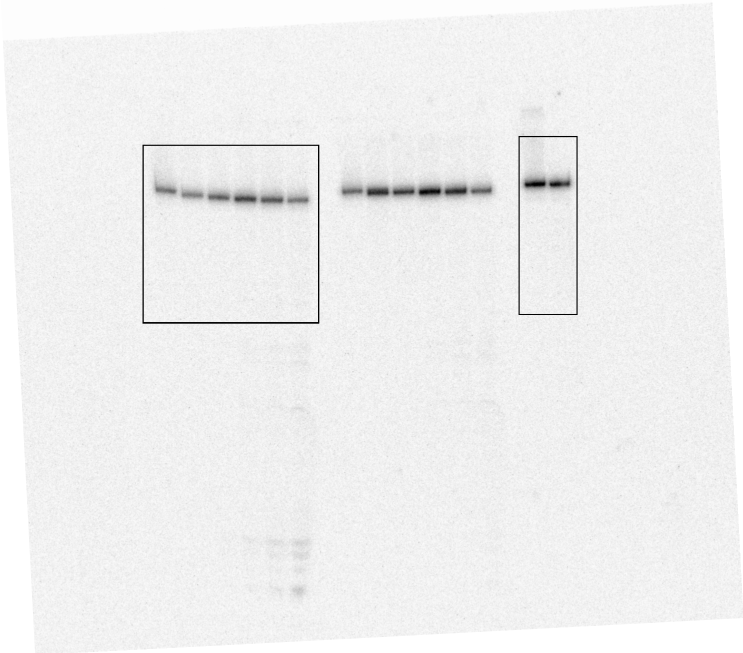

Sup. Figure 2A

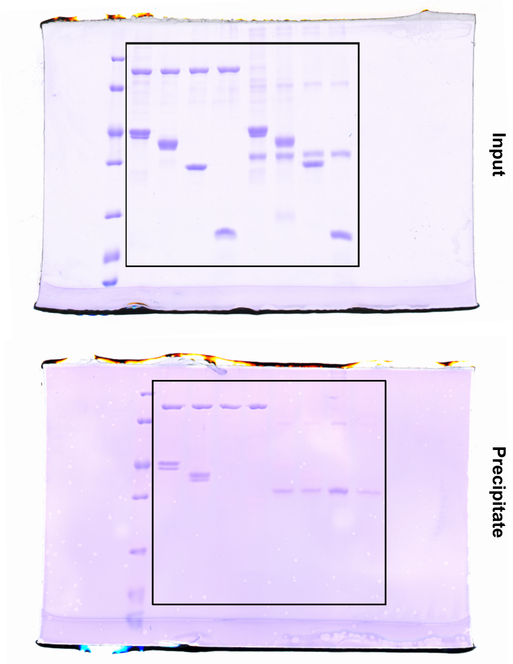

Sup. Figure 3B

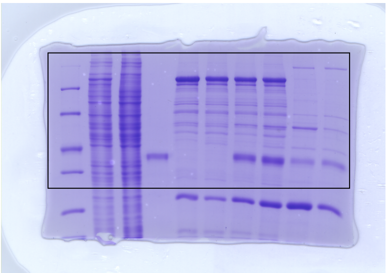

Sup. Figure 3C

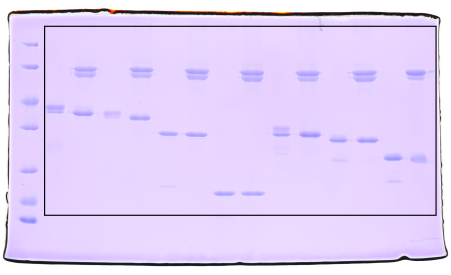

Sup. Figure 3D

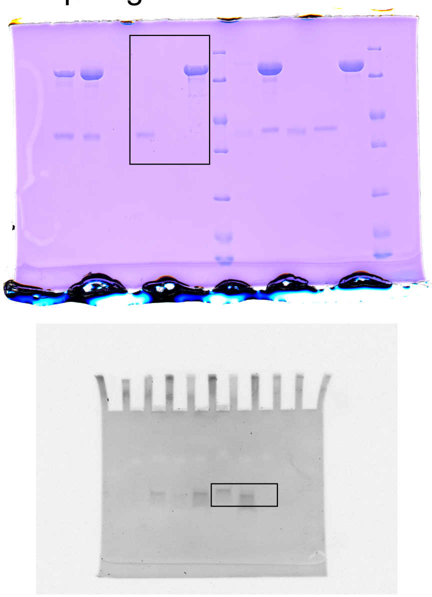

Sup. Figure 3E

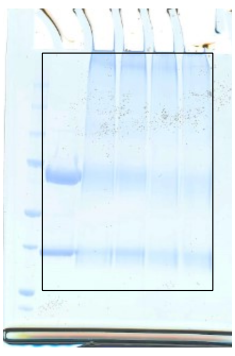

Sup. Figure 3D

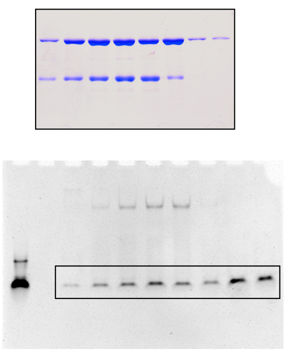

Sup. Figure 4A

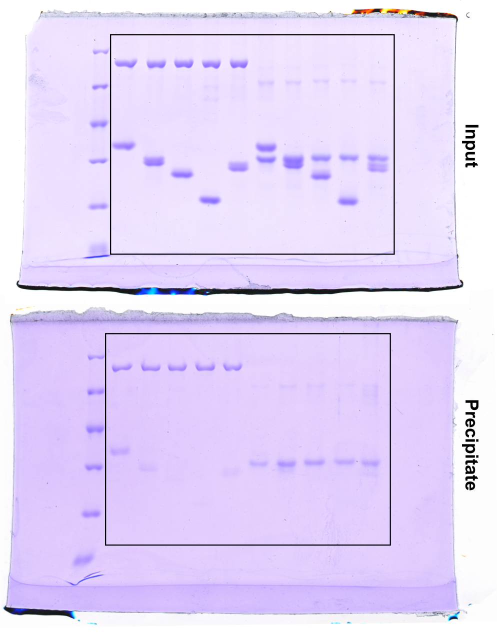

Sup. Figure 4B

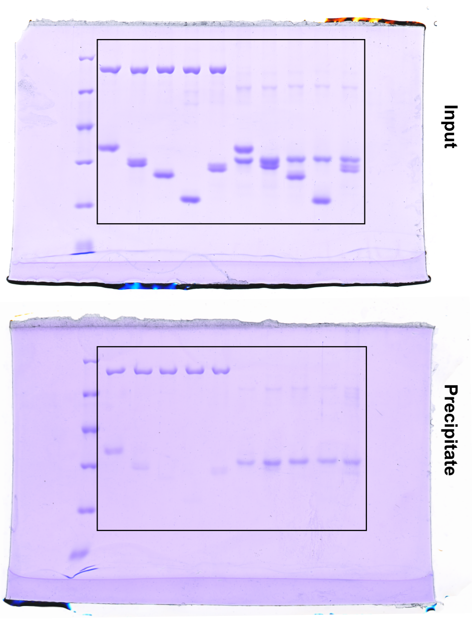

Sup. Figure 5C

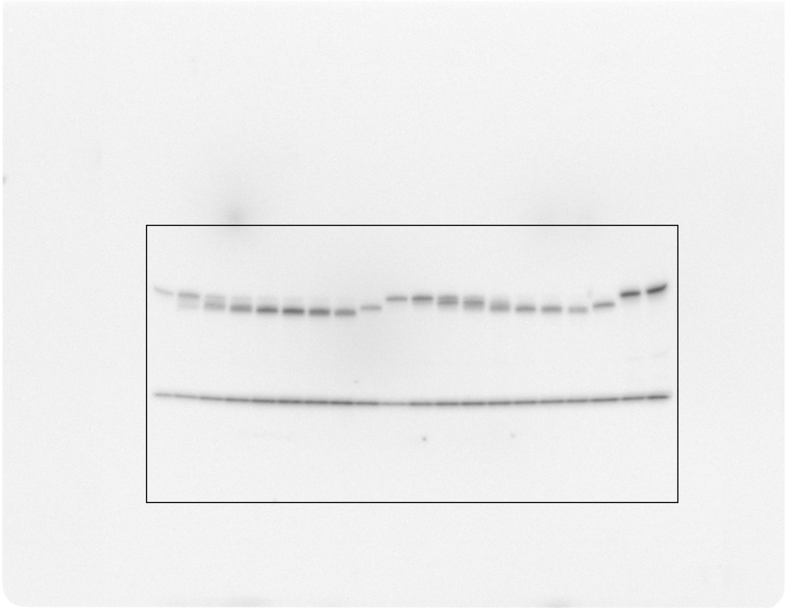

Sup. Figure 6A

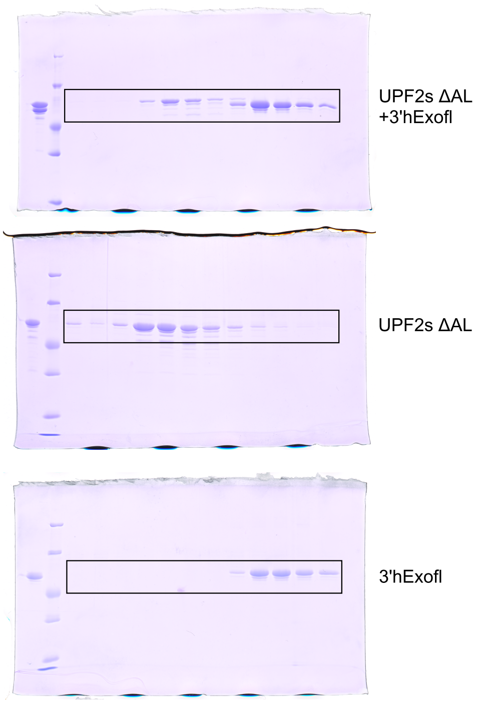

Sup. Figure 6B

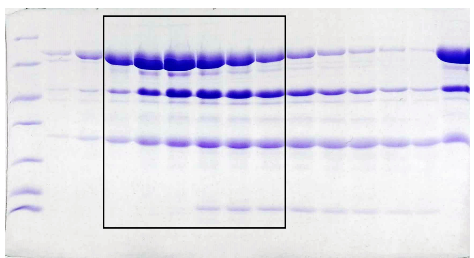

Supplement: Supplementary file 4 — Source Data [file 41467_2025_67991_MOESM4_ESM.zip › Sourcedata_Supplementary.pdf]
